# Synergistic Anticandidal Activities of Greenly Synthesized ZnO Nanomaterials with Commercial Antifungal Agents against Candidal Infections

**DOI:** 10.3390/mi14010209

**Published:** 2023-01-14

**Authors:** Mohamed Taha Yassin, Abdallah M. Elgorban, Abdulaziz A. Al-Askar, Essam Nageh Sholkamy, Fuad Ameen, Khalid Maniah

**Affiliations:** Botany and Microbiology Department, College of Science, King Saud University, P.O. Box 2455, Riyadh 11451, Saudi Arabia

**Keywords:** green synthesis, *Camellia sinensis*, terbinafine, antifungal, synergism

## Abstract

The high occurrence of mycological resistance to conventional antifungal agents results in significant illness and death rates among immunodeficient patients. In addition, the underprivileged therapeutic results of conventional antifungal agents, besides the potential toxicity resulting from long term therapy necessitate the fabrication of efficient antimicrobial combinations. Hence, the objective of the present investigation is to synthesize, characterize and investigate the anticandidal action of green zinc oxide nanoparticles (ZnO-NPs) formulated using *Camellia sinensis* leaf extract against three candidal pathogens. The eco-friendly synthesized ZnO-NPs were characterized utilizing different physicochemical methods and their anticandidal potency was tested utilizing a disk diffusion assay. In this setting, the size of the biofabricated ZnO-NPs was detected using transmission electron microscope (TEM) micrographs, recording an average particle size of 19.380 ± 2.14 nm. In addition, zeta potential analysis revealed that the ZnO-NPs surface charge was −4.72 mV. The biogenic ZnO-NPs reveal the highest anticandidal activity against the *C. tropicalis* strain, demonstrating relative suppressive zones measured at 35.16 ± 0.13 and 37.87 ± 0.24 mm in diameter for ZnO-NPs concentrations of 50 and 100 μg/disk, respectively. Excitingly, *Candida glabrata* showed a high susceptibility to the biofabricated ZnO nanomaterials at both ZnO-NPs’ concentrations (50 and 100 μg/disk) compared to the control. Moreover, the biosynthesized ZnO-NPs revealed potential synergistic effectiveness with nystatin and terbinafine antifungal agents against the concerned strains. The maximum synergistic efficiency was noticed against the *C. glabrata* strain, demonstrating relative synergistic percentages of 23.02 and 45.9%, respectively. The biogenic ZnO-NPs revealed no hemolytic activity against human erythrocytes revealing their biosafety and hemocompatibility. Finally, the high anticandidal effectiveness of biogenic ZnO-NPs against the concerned candidal pathogens, as well as potential synergistic patterns with conventional antifungal agents such as nystatin and terbinafine, emphasize the prospective application of these combinations for the fabrication of biocompatible and highly efficient antifungal agents.

## 1. Introduction

*Candida* spp. is one of the furthermost common opportunistic microbes and is found to be the main cause of 90–100% of mucosal infections [1]. It is also the fourth most common cause of hospital acquired infections, including candidemia and invasive candidiasis, which have a high mortality rate among immunocompromised patients, reaching 35–50% [2]. Additionally, vaginal candidiasis is a prevalent complication of fungal infections in women, affecting almost 70% while 20% experience recurrent vaginal candidiasis [3]. The most common yeast pathogen, *C. albicans*, continues to cause about 50% of cases of candidiasis, including fatal nosocomial candidemias with fatality rates of up to 50% of infected individuals [4]. Numerous *Candida* species have been identified as the causative agents of human illnesses, and 90% of infections are due to *C. albicans*, *C. tropicalis*, *C. glabrata*, *C. dubliniensis*, *C. krusei* and *C. parapsilosis* [5]. Several epidemiological reports have demonstrated the high frequency of the candidal resistance to common antifungal agents. In this regard, clinical isolates of *C. glabrata* revealed a high resistance profile to fluconazole antifungal agents in different countries around the world in countries such as Belgium, Canada, Czech Republic, Germany, Korea, Ireland, Slovenia, Spain, Sweden, United Kingdom and the USA, demonstrating relative MIC values that ranged from 64–128 mg/L [6,7,8]. Furthermore, a previous study found fluconazole resistance in 63 clinical isolates of *C. albicans* due to mutations in *ERG11*, which codes for lanosterol demethylase, which is the target site of azole antifungals [9]. Additionally, a previous investigation reported the resistance of *C. glabrata* to fluconazole, terbinafine, itraconazole, and clotrimazole while *C. albicans* revealed resistance to fluconazole, nystatin and clotrimazole [10]. Based on the foregoing, the poor therapeutic outcomes of conventional antifungal agents, as well as the potential toxicity of high doses of antifungal agents, necessitated finding a new combination to minimize the cytotoxicity of antifungal agents through the synergistic improvement of the anticandidal activities and the decreased necessity for high doses [11].

Manufacturing novel materials at the nanoscale is the focus of the quickly developing field of nanotechnology [12]. In other words, the goal of nanotechnology is to formulate, design, characterize, and manipulate nanomaterials between 1 and 100 nm in size [13]. The chemical approaches for fabricating nanoparticles have a number of disadvantages, such as the high toxicity that results from harmful compounds adhering to the surface of the synthesized nanomaterials, which has a harmful impact on their use in the medical field [14]. The formulation of ZnO-NPs has been described using a multiplicity of environmentally benign methods, including microwave and ultrasonic-assisted synthesis [15]. Microbes or plant extracts are utilized in biological synthesis techniques to aid in nanoparticle synthesis [16]. Green techniques have a number of qualities that make them more significant than chemical processes [17]. The primary benefit is the potential for biosynthesized nanoparticles to be used in biomedical applications or directly in living systems [18]. This is because these nanoparticles have a lower level of toxicity than those made via physicochemical processes [19]. Other benefits could include the stabilizing properties of biocomponents employed in the manufacturing process and the production of a corona by altering the nanoparticles’ surfaces, which makes them more suited for usage in living systems [20].

A previous report indicated that *Salvia officinalis* leaf extract mediated green synthesis of ZnO-NPs with antifungal activity against *C. albicans* SC5314, *C. albicans* 4175 and *C. albicans* 5112 strains demonstrating inhibitory zones of 13, 14 and 11 mm respectively at ZnO-NPs concentrations of 1.95, 1.95 and 7.81 μg/disc, respectively [21]. Another investigation demonstrated the anticandidal potency of biogenic ZnO-NPs synthesized utilizing *Scoparia Dulcis* extract at the concentration of 500 µg/mL against *C. albicans* and *A. niger* strains, demonstrating relative suppressive zones of 7.5 ±  0.7 and 8.5 ±  0.7 mm, respectively [22]. Additionally, the bioformulated ZnO-NPs fabricated using *Thymbra Spicata* L. extract revealed anticandidal activity against the *C. albicans* ATTC 90028 strain demonstrating a relative suppressive zone of 10.2 mm [23]. Furthermore, *Acacia caesia* bark extract assisted in the green biofabrication of ZnO-NPs demonstrating relative suppressive zones of 12 and 15 mm for zinc oxide nanoparticle concentrations of 500 and 1000 µg per well, respectively [24]. *Dalbergia sissoo* fresh leaf extract mediated green bioformulation of biofabricated ZnO-NPs with antimicrobial effectiveness against *Escherichia coli*, *Staphylococcus aureus* and *Candida albicans* strains [25]. Previous studies looked at antifungal activity against *C. albicans* strains, but few investigated the synergistic anticandidal activity of these biosynthesized ZnO-NPs with standard antifungal drugs. As a result, the present investigation was achieved to estimate the anticandidal effectiveness of the bioformulated ZnO-NPs against three different etiological agents of candidiasis, and to determine the possible synergism with nystatin and terbinafine antifungal agents.

## 2. Materials and Methods

### 2.1. Preparation of C. sinensis Extract

Green tea leaves were picked from a neighborhood market in Riyadh, Saudi Arabia. The herbarium at the Botany and Microbiology department identified the plant materials that were collected. *Camellia sinensis* leaves were washed once with faucet water, then dipped thrice with distilled water before being allowed to completely dry. The dried leaves were macerated using a mechanical blender to attain a uniform green tea leaf powder for the subsequent extraction procedure. In a 500 mL Elementyer flask, 50 g of the dried macerated powder were immersed in 200 mL of distilled water before the flask was heated to 70 °C for 30 min. Subsequently, the extract was then incubated overnight over a magnetic stirrer at room temperature, after which it was filtered utilizing Whatman filter paper (1) to eradicate any impurities. Finally, the extract was preserved in the refrigerator for subsequent use at 4 °C.

### 2.2. Green Bioformulation of ZnO-NPs

The biogenic formulation of ZnO-NPs was conducted utilizing the prepared water extract of *C. sinensis* leaves which acted as a reducing agent of zinc acetate hexahydrate solution. The zinc nitrate hexahydrate salt (Zn (NO_3_)_2_·6H_2_O) of grade 98% was provided from Sigma-Aldrich, Poole, Dorset, U.K. Concisely, five milliliters of the leaf extract was added to 95 mL of 0.01 M zinc nitrate hexahydrate solution in 250 mL flasks and then the reaction mixture was incubated over a magnetic stirrer for 1 h at 70 °C. Formation of brown precipitates is considered a sign of the formation of biogenic ZnO-NPs. The precipitates were separated by centrifugation of the reaction mixture at 10,000 rpm for 10 min and discarding the supernatant. The attained precipitates were washed using distilled H_2_O for the elimination of any impurities. Finally, the precipitates were incubated in an oven at 40 °C for 8 h for subsequent characterization.

### 2.3. Phytochemical Analysis of C. sinensis Extract

Gas chromatography–mass spectrometry (GC-MS) analysis of *Camellia sinensis* leaf extract was conducted to detect the main phytoconstituents contributing to reduction, stabilizing and capping of the bio-prepared ZnO-NPs. First, the water extract of green tea was concentrated using a rotatory evaporator under reduced pressure and then the concentrated extract was dried in an oven at 60 °C and finally the extract residue was dissolved in methanol for subsequent GC-MS analysis. The phytochemical analysis was achieved using a gas chromatographic-mass spectrometer (GCMS-QP2010 Plus, Shimadzu, Japan) equipped with a VF5MS capillary column (30 m length × 0.25 mm internal diameter, 0.25 μm film thickness). The following parameters were set for the analysis: with a flow rate of 1 mL/min and pure helium (99.99%) acting as the inert carrier gas, the oven’s temperature was raised to 200 °C at a ramp rate of 6 °C/min, and the injector and detector temperatures were set at 250 °C, and the injection volume was 1μL with a split ratio of 1:50. The following adjustments were made to the spectral mass detection parameters: mass range *m/z* of 40–400 amu; 2000 V electron multiplier energy; and 70 eV high ionization potential.

### 2.4. Characterization of the Eco-Friendly Formulated ZnO-NPs

Utilizing several analytical techniques, ZnO-NPs were physically and chemically characterized. Concisely, UV-Vis spectroscopy was utilized to initially confirm the formulation of the biogenic ZnO-NPs through determination of the optical spectrum in wavelengths ranging from 200 to 800 nm. Fourier transform infrared spectroscopy (FTIR) analysis was achieved to determine the primary functional groups accountable for the reduction, biostabilization and capping of these nanomaterials. In addition, the size and shape of ZnO-NPs were identified using a transmission electron microscope (JEOL, JEM1011, Tokyo, Japan), which produced high-resolution two-dimensional images at 100 kv voltage, while the elemental diagraming of ZnO-NPs was performed utilizing an energy dispersive X-ray (EDX) investigation. Additionally, X-ray powder diffraction (XRD) examination was utilized to investigate the produced samples’ crystalline nature and size. Finally, the net surface charge and the average hydrodynamic radius of the biogenic ZnO-NPs were determined using a Zeta sizer instrument (Malvern Instruments Ltd.; zs90, Worcestershire, UK).

### 2.5. Anticandidal Efficacy of ZnO-NPs

The anticandidal effectiveness of ZnO-NPs was evaluated against three candidal strains, namely *C. albicans* (ATCC 29213), *C. glabrata* (ATCC 25922), and *C. tropicalis* (ATCC 33592) using the standard disk diffusion method [26]. Initially, the candidal suspension was processed by picking up candidal colonies from 24 h fresh candidal cultures utilizing a sterile loop and immersing them into sterile saline solution (0.85%). The microbial suspension turbidity was adjusted using 0.5 McFarland standards to achieve a viable cell count of 10^6^ CFU/mL. Fresh Mueller Hinton agar (MHA) medium (Oxoid, Ltd., Hampshire, UK) supplemented with 0.5 µg/mL of methylene blue and 2% glucose was processed and dispensed in sterile Petri dishes. Afterwards, 0.5 mL of the prepared fungal suspension was spread over the poured MHA plates utilizing sterilized swabs. After dissolving the dried ZnO-NPs in methanol and sonicating them to ensure total solubility, 8 mm sterile filter paper disks were filled with 50 and 100 μg of the solubilized ZnO-NPs. Terbinafine, a standard antifungal agent, was acquired from Sigma-Aldrich, MO, USA, and 8 mm disks were saturated with 30 μg terbinafine and utilized as positive controls while filter paper disks impregnated with only methanol were utilized as negative controls. Finally, the plates were preserved in a refrigerator at 4 °C for 2 h to enable the diffusion of ZnO-NPs before being preserved in an incubator at 37 °C overnight. The inhibitory zone diameters were determined using a Vernier caliper. In order to determine the lowest concentration of ZnO-NPs exhibiting fungicidal action, the minimum inhibitory concentration (MIC) was detected against the *C. tropicalis* strain, which showed the maximum sensitivity to ZnO-NPs, utilizing broth microdilution technique as described in CLSI document M27-Ed4 [27]. The minimum fungicidal concentration (MFC) was detected by streaking from MIC concentrations with no apparent fungal growth over fresh MHA plates then incubated at 35 ± 2 °C for 48 h. The plates were monitored for candidal growth and the least concentration that revealed no fungal growth was recorded as MFC.

### 2.6. Synergistic Pattern of ZnO-NPs with Antifungals

The possible synergism between the bio-prepared ZnO-NPs and antifungal agents was investigated using a disc diffusion assay against the tested candidal strains as reported previously [28,29]. Nystatin and terbinafine standards were purchased from Sigma-Aldrich, MO, USA. Sterile filter paper disks were impregnated with the MIC concentration of the biofabricated ZnO-NPs (20 μg/mL) and the other group of disks were loaded with methanol as negative controls. Additionally, 8 mm disks were saturated with 20 and 30 μg of the antifungals nystatin and terbinafine as positive controls, respectively. Finally, filter paper disks were impregnated with antifungal agents plus the MIC concentration of ZnO-NPs (20 μg/mL) to detect the possible synergistic activity. The loaded disks were positioned over MHA plates inoculated with the microbial suspension as described before. Subsequently, the plates were reserved for 2 h in a refrigerator to permit ZnO-NPs diffusion, and then incubated at 35 ± 2 °C overnight. The inhibitory zones were estimated using Vernier caliper to measure the synergistic percentage (%) as follows:

Synergistic percentage (%) = $\frac{B–A}{A}$, whereas A and B are the diameters of suppressive zones for antifungal agent and antifungal + ZnO-NPs, respectively.

### 2.7. In Vitro Biocompatibility Assay

Fresh blood sample was centrifuged for 7 min at 12,000 rpm for the removal of red blood cells (RBCs), and then the supernatant was thrown away and the pellet was washed thrice using phosphate-buffered saline (PBS). Afterwards, the 200 μL of erythrocytes were suspended in 9.8 μL of PBS (pH: 7.2) for the formation of a PBS-erythrocyte suspension. Different concentrations of ZnO-NPs (50, 100, 200, 400, and 500 μg/mL) were mixed with erythrocytes in Eppendorf tubes and then incubated at 35 °C for 1 h. The mixture was centrifuged for 5 min at 1000 rpm then the supernatant was transferred to a 96-well plate, and the absorbance was recorded at 540 nm using a microplate (ELISA) reader. Triton X-100 (0.5%) was utilized as a positive control, showing 100% lysis, whereas PBS buffer was utilized as a negative control, showing no hemolysis. The percentage of hemolysis was detected according to the following formula:

$$\mathrm{Hemolysis}\;(\%)=(\frac{Absorbance\;of\;sample-negative\;control\;absorbance}{Positive\;control\;absorbance-negative\;control\;absorbance})\;$$

### 2.8. Cytotoxicity Assay

The most often used technique for evaluating cell metabolic activity is the methylthiazolyl diphenyl-tetrazolium bromide (MTT) test. It is a quantitative and a colorimetric assay that uses the ability of the cellular mitochondrial dehydrogenase enzyme to cleave the yellow water soluble MTT to generate insoluble dark blue/purple formazan deposits in living cells. The cytotoxic activity of the biosynthesized ZnO-NPs of concentrations 25, 50, 100, 200, 400, 800 and 1600 µg/mL was evaluated using the MTT assay against the WI-38 (normal lung fibroblast cells) cell line. A 96-well tissue culture plate was inoculated with 1 × 10^5^ cells/mL (100 µL/well) and incubated for 24 h at 37 °C. After that, the growth medium was decanted. The examined ZnO-NPs were maintained in Roswell Park Memorial Institute (RPMI) medium with 2% serum in two-fold dilutions. Each well was treated with 0.1 mL of each dilution, and three wells served as controls, receiving only serum. Finally, the plate was incubated at 37 °C. The MTT solution (5 mg/mL in PBS) was prepared and each well received 8–20 μL of MTT solution, which was well agitated for 5 min before being incubated (37 °C, 5% CO_2_) for 4 h until formazan formation. The formazan was then resuspended in 200 μL DMSO and carefully shaken for 5 min. At 560 nm the absorbance was measured, and the absorbance corresponding to the concentration that inhibited cell viability by 50% (IC_50_) was determined.

### 2.9. Statistical Analysis

The data of the current investigation was analyzed using GraphPad Prism version 8.0 (GraphPad Software, Inc., La Jolla, CA, USA) via one-way analysis of variance and Tukey’s test. All experimentations were done in triplicate ± standard error. In addition, the XRD pattern and particle size distribution histogram were plotted using OriginPro 2018.

## 3. Results and Discussion

### 3.1. Green Biofabrication of ZnO Nanoparticles

**Figure 1:**
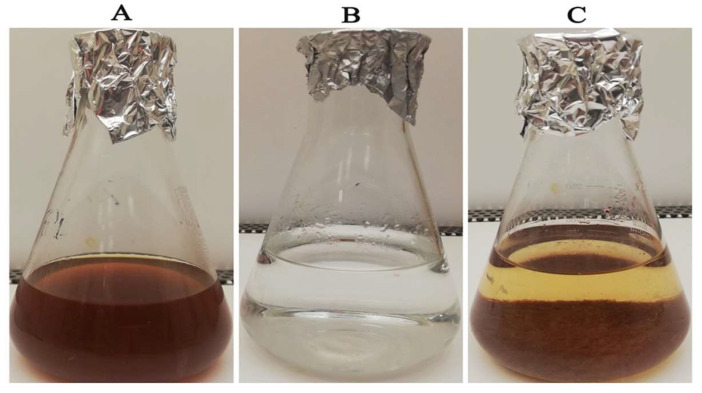
Green synthesis of biogenic ZnO nanoparticles utilizing *Camellia sinensis* aqueous leaf extract.

**Figure 2:**
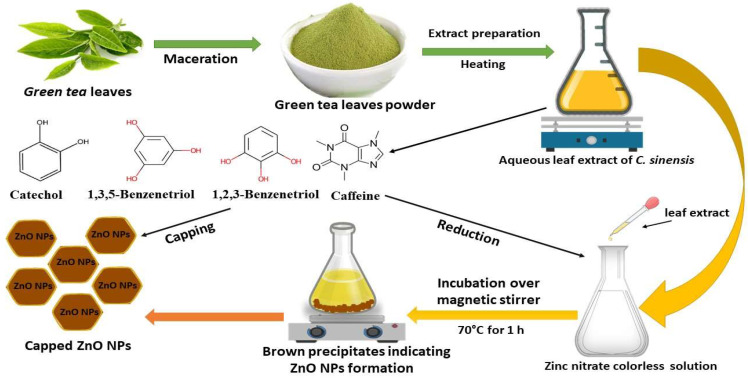
Schematic illustration of ZnO-NPs synthesis utilizing an aqueous extract of *C. sinensis* leaves.

The reduction process was conducted utilizing *C. sinensis* water extract for the bio-preparation of ZnO-NPs. Figure 1A showed the *C. sinensis* leaf extract which was utilized in the bioreduction of the colorless zinc nitrate solution (Figure 1B) causing the precipitation of brown reduced precipitates (Figure 1C), indicating the formation of ZnO-NPs. The main phytochemical components of the green tea extract were examined utilizing GC-MS analysis for the recognition of the key phytoconstituents contributing to reducing, stabilizing and capping of the biofabricated ZnO nanoparticles [30]. Phytochemical examination showed that caffeine, 1,3,5-benzenetriol, 1,2,3-benzenetriol, catechol, 1,1′-Biphenyl, 2-ethyl-, 6-Hydroxy-4,4,7a-trimethyl-5,6,7a-tetrahydrobenzofuran, and methoxy resorcinol were the main phytoconstituents of green tea extract which were thought to act as a reducing, biostabilizing and capping agents (Table 1). Our findings were in agreement with those of an earlier study, which showed that caffeine, 1,2,3-benzenetriol and 1,3,5-benzenetriol in green tea extract operationalized as a reducing and capping agent for biogenic iron nanoparticles [31]. Another study found that the phytoconstituents such as caffeine, catechol, 1,2,3-benzenetriol, methoxy resorcinol, ethanone, and 1,3,5-benzenetriol acted as capping and reducing agents during the formulation of bimetallic Fe/Pd nanoparticles using an aqueous green tea extract [32]. Accordingly, the detected biomolecules in green tea extract such as catechol, caffeine, 1,2,3-benzenetriol and 1,3,5-benzenetriol served as reducing agents of zinc nitrate solution for the successful formation of biogenic ZnO nanoparticles (Figure 2).

### 3.2. UV Spectral Analysis

**Figure 3:**
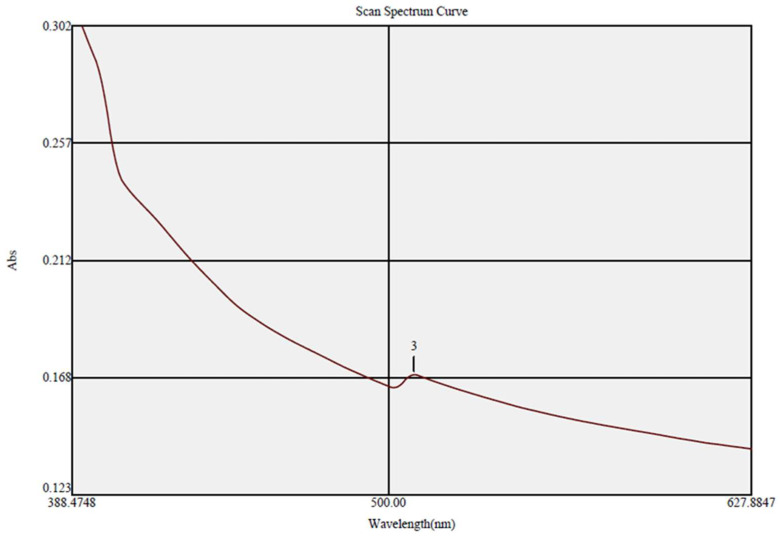
UV spectrum of the green bio-prepared ZnO-NPs.

The biofabricated ZnO-NPs were examined utilizing a UV spectrophotometer, demonstrating the formation of the characteristic absorption peak at 509 nm, which shows the surface plasmon resonance of the biofabricated ZnO-NPs (Figure 3). Our findings were in line with that of a prior study which established the green bio-preparation of pure ZnO-NPs using *Punica granatum* peels, demonstrating a UV emission peak at 509 nm [33]. The band gap energy of ZnO-NPs was appraised according to Planck’s equation: *E_g_ = hc/λ,* whereas *c* is the speed of light = 3.0 × 10^8^ m s^−1^, *h* is Planck constant (4.136 × 10^−15^ eV s), and *λ* is the wavelength (509 × 10^−9^ m). The band gap energy of the eco-friendly prepared ZnO-NPs was determined to be 3.4 eV, and this finding was consistent with earlier findings [34].

### 3.3. Transmission Electron Microscope (TEM) Examination

**Figure 4:**
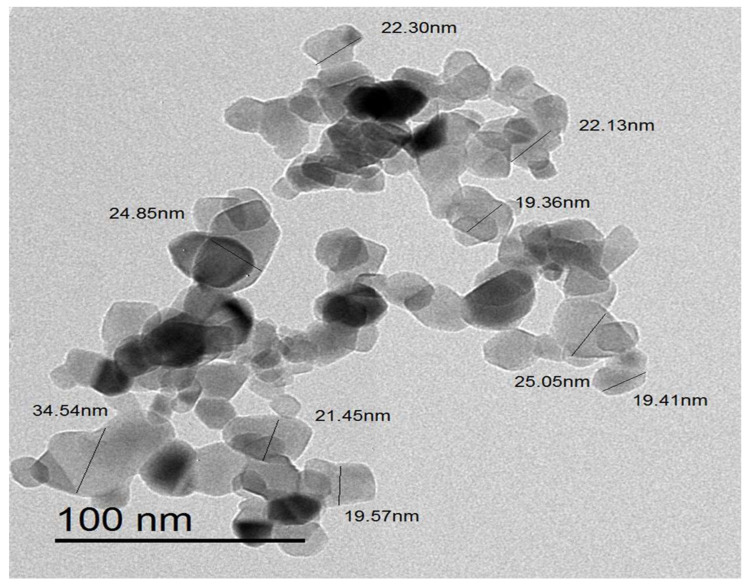
TEM micrograph of the biosynthesized ZnO nanoparticles.

**Figure 5:**
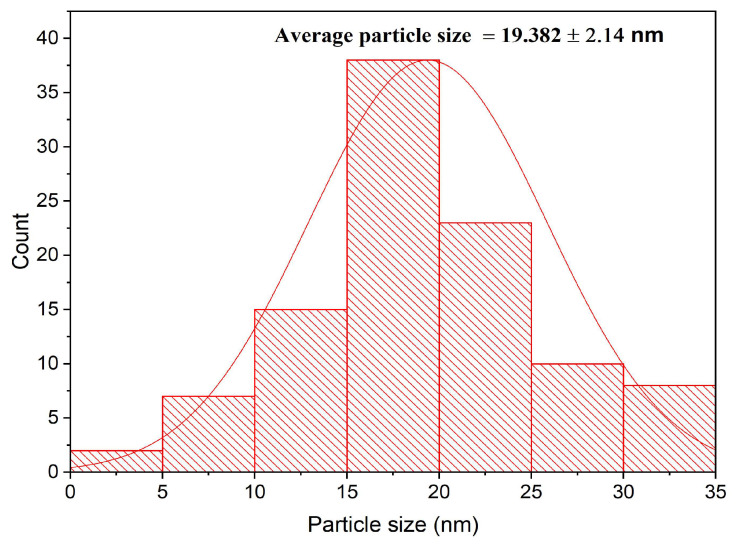
Particle size distribution histogram of the biofabricated ZnO nanoparticles.

Using TEM examination, the morphological traits of ZnO-NPs were examined. The hexagonal shape of ZnO-NPs was confirmed by TEM micrographs, which also revealed that the particle size ranged from 5 to 35 nm (Figure 4). The ZnO-NPs had a median particle size diameter of 19.380 ± 2.14 nm, as demonstrated in the particle size distribution histogram (Figure 5). Our results were in consistent with that of the previous literature which confirmed the hexagonal shape of the bio-prepared ZnO-NPs [35]. Interestingly, the size diameter of the bio-prepared ZnO-NPs was lower than that reported in recent research, which showed that fresh and dry alhagi plant were employed to bioformulate ZnO-NPs with size diameter ranging from 25 to 100 nm and 35 to 100 nm, respectively [36]. On the other hand, our findings were also at odds with those of a previous report, which proved that the bio-preparation of spherical ZnO-NPs utilizing *Pandanus odorifer* leaf extract and demonstrated that the nanosize of ZnO-NPs was 90 nm in diameter [37]. Additionally, as previously reported, a TEM investigation of ZnO-NPs formulated utilizing *Andrographis serpilifolia* leaf extract showed that the mean particle size diameter was 80 nm [38]. Collectively, the small size of the bio-prepared ZnO-NPs fabricated utilizing *C. sinensis* aqueous leaf extract in the present study compared to recent previous reports indicate the high efficacy of the eco-friendly biosynthesis route using green tea extract for biofabrication of small sized ZnO-NPs.

### 3.4. Energy-Dispersive X-ray (EDX) Examination

**Figure 6:**
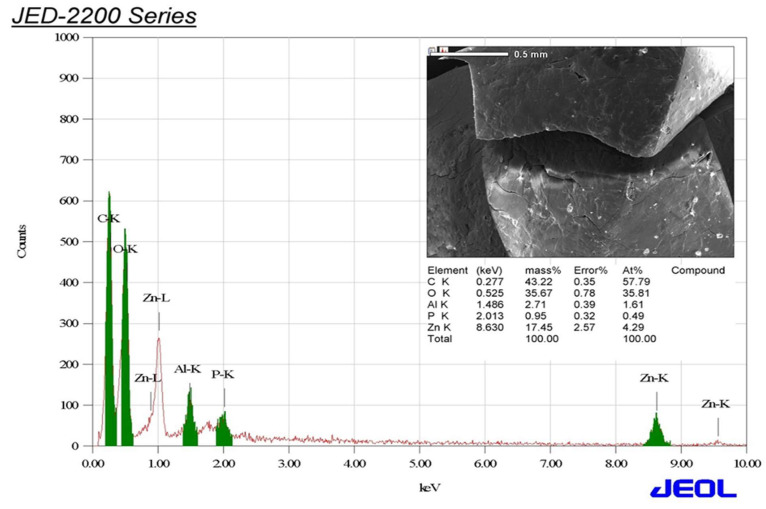
EDX analysis of the biofabricated ZnO-NPs using green tea extract.

The elemental outline of ZnO-NPs shows the presence of the elements zinc, oxygen, carbon, aluminum, and phosphorous with relative mass percentages of 17.45, 35.67, 43.22, 2.71 and 0.95%, respectively (Figure 6). EDX analysis indicated the peaks related to the optical absorption of the bioformulated ZnO nanoparticles. In this regard, the peaks detected at 0.5, 1.1 and 8.6 keV corresponded to O *Kα*, Zn *Lα*, Zn *Kα*, and Zn *Kβ*, respectively [39]. Accordingly, EDX analysis confirmed the successful biosynthesis of ZnO nanoparticles. The remaining peaks of aluminum, phosphorus, and carbon could be attributed to the green tea extract’s capping biomolecules as stated in previous literature [40].

### 3.5. Fourier Transform Infrared Spectroscopy (FTIR) Examination

**Figure 7:**
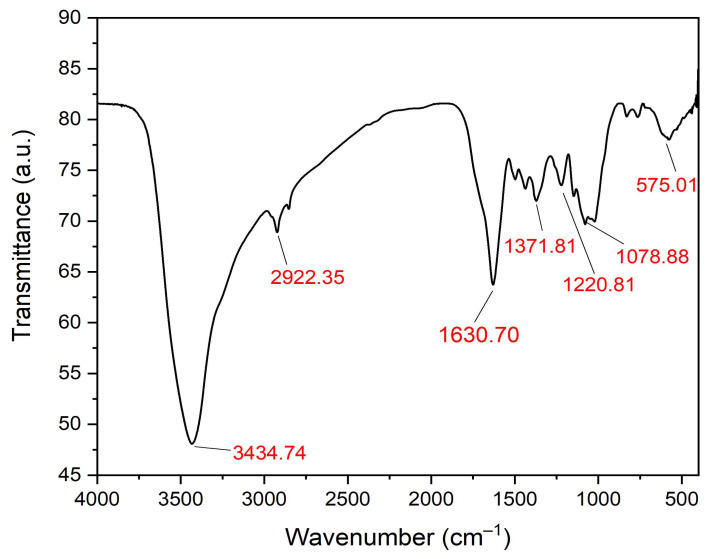
FTIR spectrum of the eco-friendly formulated ZnO-NPs.

The ZnO-NPs’ functional groups were identified utilizing the FTIR investigation to recognize the biomolecules functionalized as capping, reduction, and stabilizing agents. The FTIR spectrum demonstrated the presence of different absorption peaks at 3434.74, 2922.35, 1630.70, 1371.81, 1220.18, 1078.88 and 575.01 cm^−1^ (Figure 7). The broad absorption peak noticed at 3434.74 cm^−1^ was accredited to the phenolic biomolecules of green tea extract, while the peak observed at 2922 cm^−1^ was allocated to the C–H stretching of alkanes (Table 2). Furthermore, the C=C stretching of the alkenyl functional group was detected owing to the peak observed at 1630.70 cm^−1^. Furthermore, the weak band detected at 1371.81 cm^−1^ was allotted to C=C stretching of carboxylic acids capped on ZnO-NPs. The tiny band observed at 1220.18 cm^−1^ was attributed to the OH bending vibrational mode from phenolic or alcoholic functional groups. On the other hand, the narrow peak observed at 1078.88 cm^−1^ was attributed to the C–N stretching of aliphatic amines. Finally, the weak band at 575.01 cm^−1^ was attributed to the vibrational mode of Zn-O stretching.

### 3.6. XRD Investigation of ZnO-NPs

**Figure 8:**
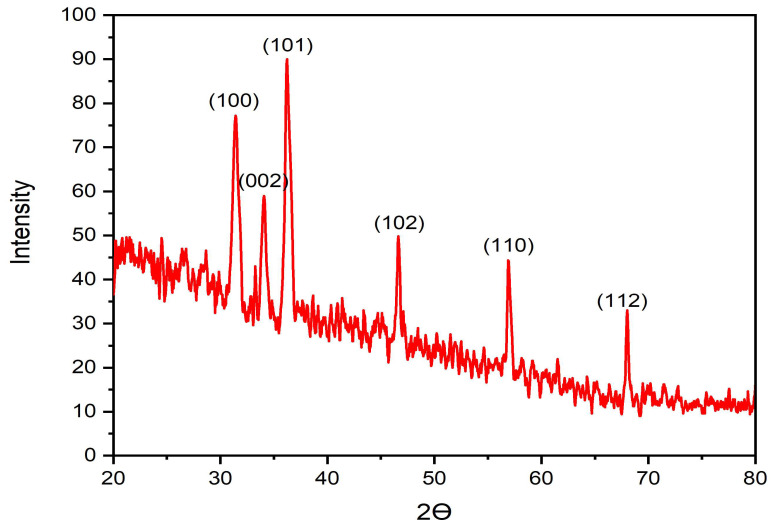
XRD configuration of the bio-prepared ZnO-NPs.

The crystalline characteristics of the bio-prepared ZnO-NPs were inspected using an XRD examination. The XRD pattern showed diffraction peaks at 2 theta degrees of 31.46, 34.06, 36.18, 46.62, 56.86, and 68.02° (Figure 8). These peaks are reflective of (100), (002), (101), (102), (110), and (112) crystal planes of ZnO-NPs, respectively (JCPDS No: 36-1451) [41]. These findings confirmed the hexagonal wurtzite structure of the bio-prepared ZnO-NPs as demonstrated in previous studies [42]. Scherrer’s equation (crystalline size = kλ/β cos θ) was applied to detect the crystalline size of ZnO-NPs, where k is the Scherrer’s constant (K = 0.94), β is the full width at half maximum (FWHM) of the most intense diffraction peak at 36.18° which is estimated to be 0.5017, and λ is the X-ray wavelength (1.54178 Å). Accordingly, the crystalline size was estimated to be 17.41 nm and this finding agrees with TEM analysis. Our findings were in line with that of a prior study which specified the recognition of seven diffraction peaks at 2θ angles of 31.78, 34.44, 36.28, 47.55, 56.62, 62.83 and 67.96°, which were correlated to crystal planes of (100), (002), (101), (102), (110), (103) and (112), respectively, and the corresponding crystalline size was estimated to be 17.47 nm [43].

### 3.7. Zeta Analysis of the Bio-Prepared ZnO-NPs

**Figure 9:**
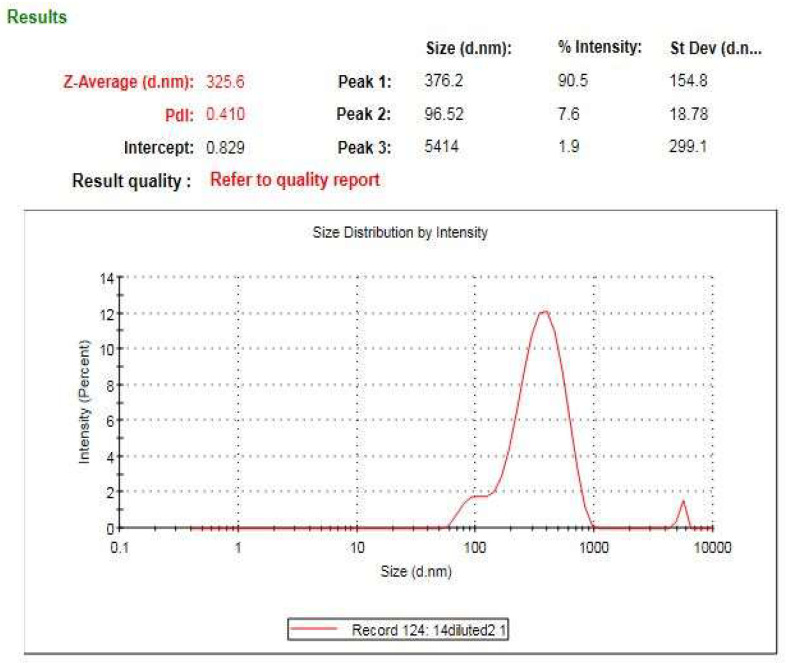
Dynamic light scattering of the bio-prepared ZnO-NPs.

**Figure 10:**
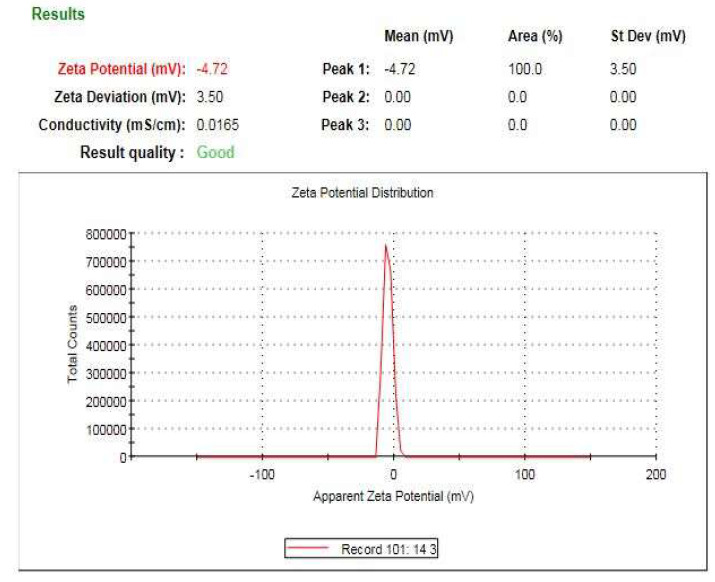
Zeta potential investigation of the bio-prepared ZnO-NPs.

The average hydrodynamic diameter of the bio-prepared ZnO-NPs was assessed using a dynamic light scattering technique [44]. Moreover, the surface charge of ZnO-NPs was appraised using zeta potential examination. The hydrodynamic diameter of ZnO-NPs was determined to be 325.6 nm (Figure 9) which was larger than that detected by TEM and XRD investigations due to the adsorption of the capping biomolecules on the ZnO-NPs’ surface [45]. Additionally, the dispersant-particle hydrodynamic size interference, which comprises the estimation of both particle diameter and their surrounding diffusion layer, could be the cause of the significant disparity in particle size between the DLS approach and both TEM and XRD examinations [46]. The estimated surface charge of the bio-prepared ZnO-NPs was estimated to be −4.72 mV (Figure 10) and this finding was in agreement with that of a previous investigation [47]. The surface charge of the elements is activated during biological interactions by nanoparticles with a zeta potential value between −10 and +10 mV, which was proven by previous reports [48,49].

### 3.8. Screening of Anticandidal Efficiency of Green Biofabricated ZnO-NPs

**Figure 11:**
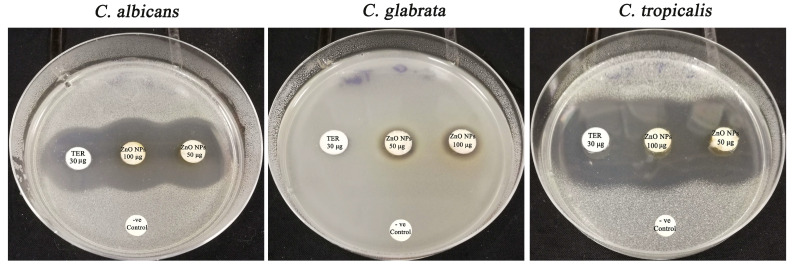
Antibiogram of the eco-friendly formulated ZnO-NPs against the concerned fungal strains.

**Figure 12:**
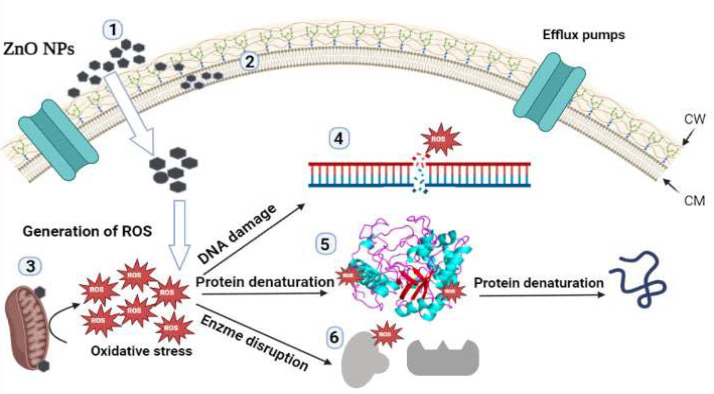
Proposed mechanism of anticandidal action of the biosynthesized ZnO-NPs. The diagram showed the mechanistic anticandidal pathways of the biosynthesized ZnO-NPs: 1—Zinc oxide nanoparti-cles and Zn^2+^ accumulation over the cell wall of candidal cells results in formation of pores which in turn leads to leakage of intracellular constituents. 2—The biosynthesized ZnO-NPs attack the lipid bilayer of candidal cell membrane resulting in disruption of membrane potential. 3—The nano-particles adhered to the mitochondrial membrane resulting in increasing the oxidative stress and generation of reactive oxygen species (ROS). 4—The generated ROS resulted in destroying cellular DNA. 5—The ROS disrupted the biological macromolecules of fungal cell as proteins resulting in their denaturation. 6—Furthermore, ROS mediated destruction of the enzymatic system of the microbial cell. (ZnO-NPs: zinc oxide nanoparticles; ROS: reactive oxygen species; CW: cell wall; CM: cell membrane).

The biofabricated ZnO-NPs were examined for their antifungal potency against the concerned fungal strains utilizing the standard disk diffusion method (Figure 11) [29]. By the way, the biofabricated ZnO-NPs at the concentration of 50 μg/disk displayed antifungal action against *C. albicans*, *C. tropicalis* and *C. glabrata* strains, and inhibitory zones ranged from 12.78 to 35.16 nm (Table 3). The antifungal effectiveness data were higher than those from a previous study which showed the facile green bio-preparation of ZnO-NPs using *Azadirachta indica* (L.) extract, demonstrating inhibition zone diameters at the concentration of 50 μg/disk measuring 17.6 ± 0.76 and 14.1 ± 0.28 mm against *C. albicans* and *C. tropicalis* strains, respectively [50]. Additionally, the suppressive zone of the green bio-prepared ZnO-NPs utilizing *Murraya koenigii* against the *C. tropicalis* strain was found to be 7.0 ± 0.50 mm, which was significantly lower than the inhibitory zone reported in our current investigation at the same concentration of 100 μg/disk of 37.87 ± 0.24 mm [51]. This could be explained by the small particle size of the prepared ZnO-NPs (19.832 ± 2.14 mm) compared to that of the previous report in which it was detected to be 22 nm. Smaller particles typically have higher surface-to-volume ratios than larger ones, making them more effective agents for anticandidal applications [52]. Collectively, the high potency of the eco-friendly bio-prepared ZnO-NPs against fungal strains asserted the high efficiency of the green method of ZnO-NPs’ biosynthesis using *C. Sinensis* extract. The suggested mode of action of the bio-prepared ZnO-NPs is illustrated in Figure 12. The biogenic ZnO-NPs exert their fungicidal effects via a multiplicity of mechanisms, including the destruction of cellular components (such as cell walls, membranes, and other cellular organelles), the impairment of essential biological macromolecules (such as enzymes and proteins), the distraction of DNA replication, and the generation of reactive oxygen species (ROS), which interfered with the anti-oxidative system [53]. The biofabricated ZnO-NPs were inspected for their minimum inhibitory concentration (MIC) and minimum fungicidal concentration (MFC) against the *C. tropicalis* strain which proved the maximum sensitivity to the bio-prepared ZnO-NPs. In this regard, the MIC of biofabricated ZnO nanomaterials against the *C. tropicalis* strain was detected to be 20 μg/mL while the MFC was detected to be 40 μg/mL.

### 3.9. Detection of Synergistic Efficacy of the Bio-Prepared ZnO-NPs with Antifungals

**Figure 13:**
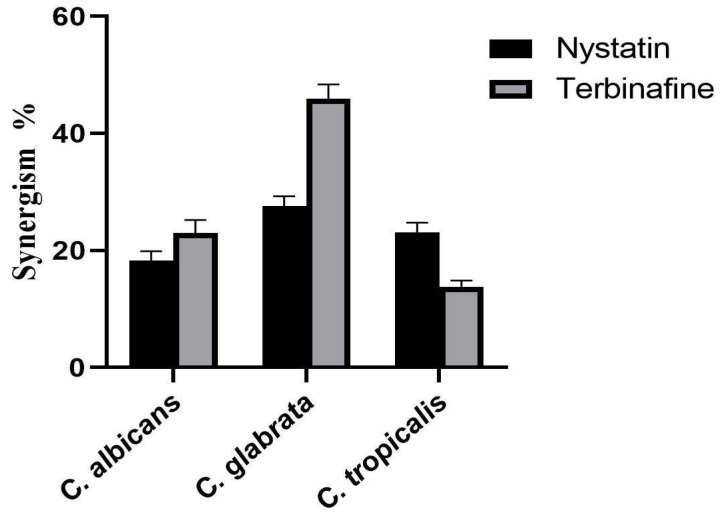
Synergistic proportions (%) of the eco-friendly prepared ZnO-NPs with nystatin and terbinafine antifungals against *Candida* strains.

The biofabricated ZnO-NPs were examined for their possible synergism with terbinafine and nystatin antifungal agents against candidal strains. When compared to nystatin, terbinafine shows greater synergistic efficacy against *C. albicans* and *C. glabrata* strains, with relative synergism percentages of 23.02 and 45.9%, respectively (Figure 13). On the other hand, the bio-prepared ZnO-NPs demonstrate better synergistic efficacy with nystatin against *C. tropicalis* than with the antifungal terbinafine, displaying synergism percentages of 23.14 and 13.81%, respectively.

The synergistic impact of ZnO-NPs with antifungal agents such as nystatin and terbinafine was accredited to both ZnO-NPs and antifungal drugs targeted different cellular constituents. Against this background, the terbinafine antifungal agent is a member of the class of allyl amines that best exhibits fungicidal activity by obstructing ergosterol formation at its beginning stages. By reason of the squalene epoxidase enzyme’s action, which oxidizes squalene intermediates and accumulates them, ergosterol production is inhibited. This lack of ergosterol causes fungal cell death by increasing cellular permeability and upsetting cellular organization [54]. Correspondingly, we proposed that the synergistic effect of terbinafine with the bio-prepared ZnO-NPs results from the ergosterol inhibition action of terbinafine that disrupted the cellular permeability and led to the internalization of the bio-prepared ZnO-NPs that also underwent oxidation of cellular components such as DNA, protein, and enzymes leading to fungal cell death. The proposed antifungal action of ZnO nanoparticles was schematically illustrated. It was proposed that after ZnO-NPs were internalized into the fungal cell as a result of the disturbance of the cell membrane triggered by the antifungal agent terbinafine, the ZnO-NPs increased the generation of ROS, which consequently disrupted the biological macromolecules of the fungal cell such as DNA, protein, and enzymes, and ultimately resulted in fungal cell death.

The polyene antifungal drugs, of which nystatin is a member, are notable by their long chemical structures, which consequently permit them to form channels in the fungal cell membrane, impairing membrane permeability and causing the discharge of essential components. Cellular acidification and the cessation of enzymatic activity are initiated by the entry of hydrogen ions and the efflux of potassium ions from the fungal cell. Additionally, the cells let out leaks of carbohydrates and amino acids which finally leads to the indication of fungal cell death [55]. On the contrary, the antifungal mechanism of ZnO-NPs against candidal cells occurs through the generation of ROS, which disrupts the fungal cell’s oxidative system and oxidizes essential cellular components such as protein, DNA and enzymes, and ultimately blocking the cellular enzymatic system and preventing cellular replication and inducing cell death [56]. Based on the foregoing, we postulate that the antifungal drug nystatin reduced membrane permeability, allowing ZnO-NPs to enter the cell and disrupt DNA, protein and enzymes through the production of ROS, ultimately resulting in the fungal cell death. Collectively, the ZnO-NPs’ combinations with terbinafine and nystatin could be a promising source of highly efficient anticandidal agents owing to their synergistic potency against the concerned strains.

### 3.10. In Vitro Biocompatibility Assay

**Figure 14:**
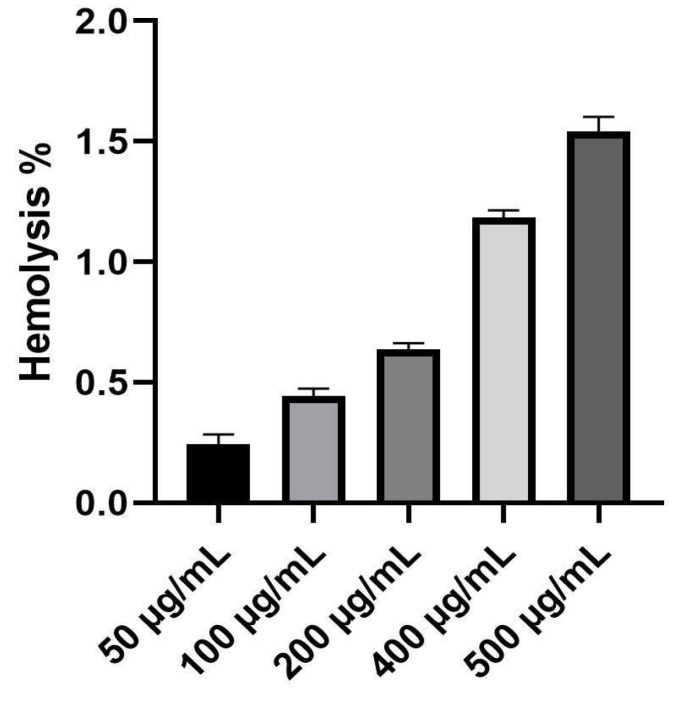
Hemolysis percentages (%) of different concentrations of the biofabricated ZnO-NPs against hRBCs.

The biocompatibility of the biofabricated ZnO-NPs with human red blood cells (hRBCs) was evaluated to detect any toxicological potential of these nanomaterials. Different concentrations of the biogenic ZnO-NPs formulations (50–500 μg/mL) were incubated with the freshly prepared RBCs. As seen in Figure 14, the biogenic ZnO-NPs revealed hemolytic percentages of 0.24, 0.44, 0.63, 1.18 and 1.54% for ZnO-NPs’ concentrations of 50, 100, 200, 400 and 500 μg/mL, respectively. The hemolytic assay data confirmed that the bioformulated ZnO-NPs exhibit no hemolytic activity even at the highest concentrations, demonstrating their biosafety and hemocompatibility.

### 3.11. Cytotoxicity Assay

**Figure 15:**
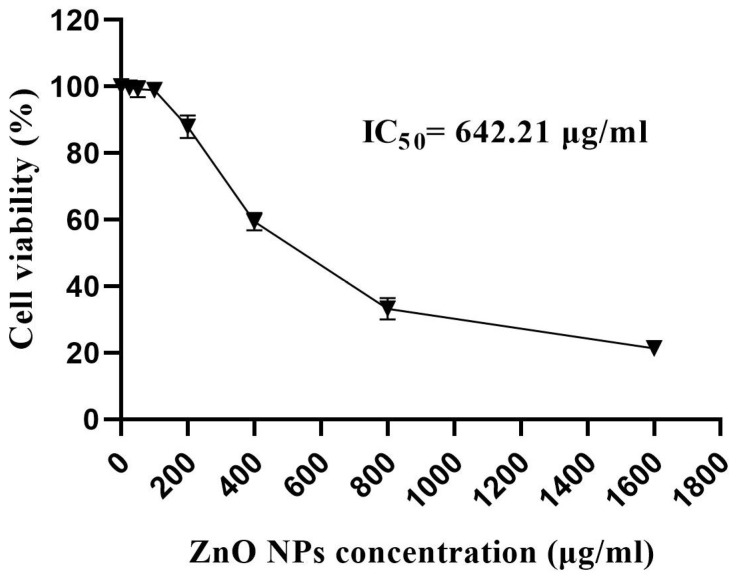
Cell viability percentage (%) of WI-38 cells treated with different concentrations of ZnO-NPs.

The cytotoxic impact of ZnO-NPs was examined against normal lung fibroblast cells to confirm their biosafety. In this regard, the cytotoxic effect of different concentrations of ZnO-NPs was evaluated using an MTT assay against the WI-38 cell line. The biogenic ZnO-NPs revealed low cytotoxic activity against WI-38 cells, demonstrating an average IC_50_ of 642.21 μg/mL (Figure 15). Our results agree with that of a prior report which specified that the bio-prepared ZnO-NPs produced *Limonium pruinosum* L. Chaz. extract and recorded a relative IC_50_ of 568.59 μg/mL against the WI-38 cell line [57]. The US Food and Drug Administration recommends that zinc oxide nanoparticles be considered safe (i.e., generally recognized as safe) [58]. Overall, the findings of the hemolytic assay test were supported by the ZnO NPs’ cytotoxicity test against WI-38 cells, confirming the biosafety of the bio-produced ZnO-NPs for different applications.

## 4. Conclusions

Camellia sinensis leaf water extract facilitated the eco-friendly biofabrication of biofabricated ZnO-NPs with unique physicochemical properties. The bio-produced ZnO-NPs showed high anticandidal activity against the tested strains, which could be consigned to the small size of ZnO-NPs, as well as the capping biomolecules of the green tea extract, which were identified using FTIR analysis. Interestingly, the biosynthesized ZnO-NPs revealed a high anticandidal activity against the *C. glabrata* strain and also reveal potential synergistic activity with nystatin and terbinafine antifungal agents. The synergistic potency of the bioformulated ZnO-NPs with antifungals emphasizes the possibility of using these nanoparticles in the bioformulation of highly efficient antimicrobial combinations while avoiding the potential toxicity associated with high antifungal doses.

## Figures and Tables

**Table 1:** GC-MS analysis of *Camellia sinensis* leaf extract.

Compounds	Chemical Formula	Chemical Structure	Mol. Weight	RT	% of Total
Catechol	C_6_H_6_O_2_	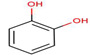	110.11	12.465	6.53
1,1’-Biphenyl, 2-ethyl-	C_14_H_14_	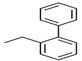	182.26	13.671	5.19
Methoxy resorcinol	C_7_H_8_O_3_	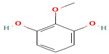	140.14	15.263	3.78
1,2,3-Benzenetriol	C_6_H_6_O_3_	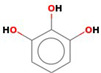	126.11	17.461	10.79
1,3,5-Benzenetriol	C_6_H_6_O_3_	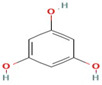	126.11	24.689	19.56
6-Hydroxy-4,4,7a-trimethyl-5,6,7a-tetrahydrobenzofuran	C_11_H_16_O_3_	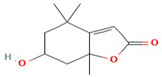	196.24	25.537	4.63
Caffeine	C_8_H_10_N_4_O_2_	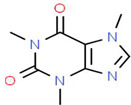	194.19	27.167	49.51

**Table 2:** Functional groups of the biofabricated ZnO-NPs synthesized using green tea extract.

No.	Absorption Peak (cm^−1^)	Appearance	Functional Groups	Molecular Motion
1	3434.74	Strong, broad	Alcohols and phenols	O–H stretching
2	2922.35	Weak	Alkanes	C–H stretching
3	1630.70	Medium	Alkenyl group	C=C stretching
4	1371.81	Weak	Carboxylic acids	C=C stretching
5	1220.18	Weak	Alcohols and phenols	OH bending
6	1078.88	Weak	Aliphatic amines	C–N stretching
7	575.01	Weak	Metal oxide bonds	Zn–O stretching

**Table 3:** Antifungal activity of the bio-prepared ZnO-NPs against *Candida* strains.

Fungal Strains	Inhibition Zone Diameter (mm)			
	ZnO-NPs (50 µg/Disk)	ZnO-NPs (100 µg/Disk)	Terbinafine (30 µg/Disk)	−ve Control
*C. albicans*	30.56 ± 0.27	32.34 ± 0.11	26.12 ± 0.09	0.00 ± 0.00
*C. tropicalis*	35.16 ± 0.13	37.87 ± 0.24	35.34 ± 0.41	0.00 ± 0.00
*C. glabrata*	12.78 ± 0.17	13.24 ± 0.15	8.65 ± 0.21	0.00 ± 0.00

## Data Availability

Not applicable.
